# Diagnosis and Surgical Management of Landzert's Paraduodenal Hernia

**DOI:** 10.7759/cureus.83575

**Published:** 2025-05-06

**Authors:** Ishan Deshmukh, Aaryan Patel, Yasmine Saikali, Haashim Rahman, Abbas Merchant, Constantino G Lambroussis

**Affiliations:** 1 Vascular Surgery, Lake Erie College of Osteopathic Medicine, Elmira, USA; 2 Physical Medicine and Rehabilitation, Lake Erie College of Osteopathic Medicine, Erie, USA; 3 Dermatology, Lake Erie College of Osteopathic Medicine, Erie, USA; 4 Anesthesiology, Lake Erie College of Osteopathic Medicine, Erie, USA; 5 Anesthesia, Lake Erie College of Osteopathic Medicine, Erie, USA; 6 Osteopathic Medicine/Family Medicine, Lake Erie College of Osteopathic Medicine, Elmira, USA

**Keywords:** acute volvulus, closed-loop small bowel obstruction, left paraduodenal hernia, paraduodenal hernia, small bowel obstruction

## Abstract

Landzert's paraduodenal hernia is a rare congenital internal hernia that results from the failure of the left mesentery to fuse completely with the parietal peritoneum during embryological development. This creates a congenital defect, the fossa of Landzert, through which small bowel loops can herniate into the retroperitoneum, leading to intermittent or acute small bowel obstruction (SBO). We present the case of a 33-year-old man with a history of episodic postprandial abdominal pain who presented with acute-onset severe abdominal pain, nausea, and vomiting. Computed tomography (CT) revealed small bowel loops herniating through the fossa of Landzert, indicative of a left paraduodenal hernia, along with a closed-loop small bowel obstruction. The patient underwent laparoscopic repair with conversion to open reduction and primary suture repair using 2-0 Stratafix (Ethicon, Raritan, NJ) sutures of the left paraduodenal hernia without having to resect any bowel segments. No intraoperative complications occurred, and the patient remains free of gastrointestinal discomfort several months post-operation.

## Introduction

Landzert's paraduodenal hernia is a rare form of internal hernia, accounting for less than 1% of all hernias. Paraduodenal hernias can be either left- or right-sided due to congenital defects, with the left-sided paraduodenal hernia being more common than the right-sided counterpart [1]. In this condition, a segment of the small intestine protrudes posteroinferiorly through the fossa of Landzert, an abnormal aperture in the peritoneum, the thin membrane that lines the abdominal cavity, near the ligament of Treitz, a structure that supports the duodenum, the first part of the small intestine [1]. The fossa of Landzert is a congenital peritoneal recess that forms due to the incomplete fusion of the descending mesocolon with the posterior parietal peritoneum during embryonic development [2,3]. This non-fusion creates a potential space to the left of the fourth part of the duodenum, just below the duodenojejunal junction. The fossa is bordered anteriorly by the inferior mesenteric vein and the ascending branch of the left colic artery and posteriorly by the parietal peritoneum. This type of hernia can lead to intestinal obstruction, which requires prompt surgical intervention to prevent serious complications. While an uncommon occurrence, as many as 5% of all intestinal obstructions are due to internal hernias and are associated with significant morbidity and mortality if not properly diagnosed and managed [4-7]. In particular, internal hernias such as the paraduodenal hernia of Landzert have been reported to have mortality rates over 50% if signs of strangulation are present [8].

The presentation of this condition can vary, with symptoms ranging from intermittent abdominal pain to complete intestinal obstruction [2]. Chronic presentations without signs of acute intestinal obstruction may present as recurring post-prandial abdominal discomfort, nausea, and vomiting [9]. These symptoms can be caused by the protruding segment of the small intestine through the fossa of Landzert, which can lead to intermittent obstruction and discomfort.

Due to the rarity of Landzert's paraduodenal hernia, we present a case report to increase awareness of this condition and highlight the diagnostic and management strategies. In particular, we discuss the role of advanced imaging modalities, such as computed tomography, in establishing the diagnosis, as well as the importance of timely surgical intervention to prevent complications and potentially life-threatening outcomes associated with this type of internal hernia.

## Case presentation

A 33-year-old man presented to the emergency room with an acute episode of epigastric and back pain, nausea, and vomiting. The patient reported experiencing intermittent upper abdominal discomfort for the past two years and has a past medical history of irritable bowel syndrome and gastroesophageal reflux disease. On physical examination, there was tenderness in the epigastric area without signs of peritoneal irritation.

**Figure 1:**
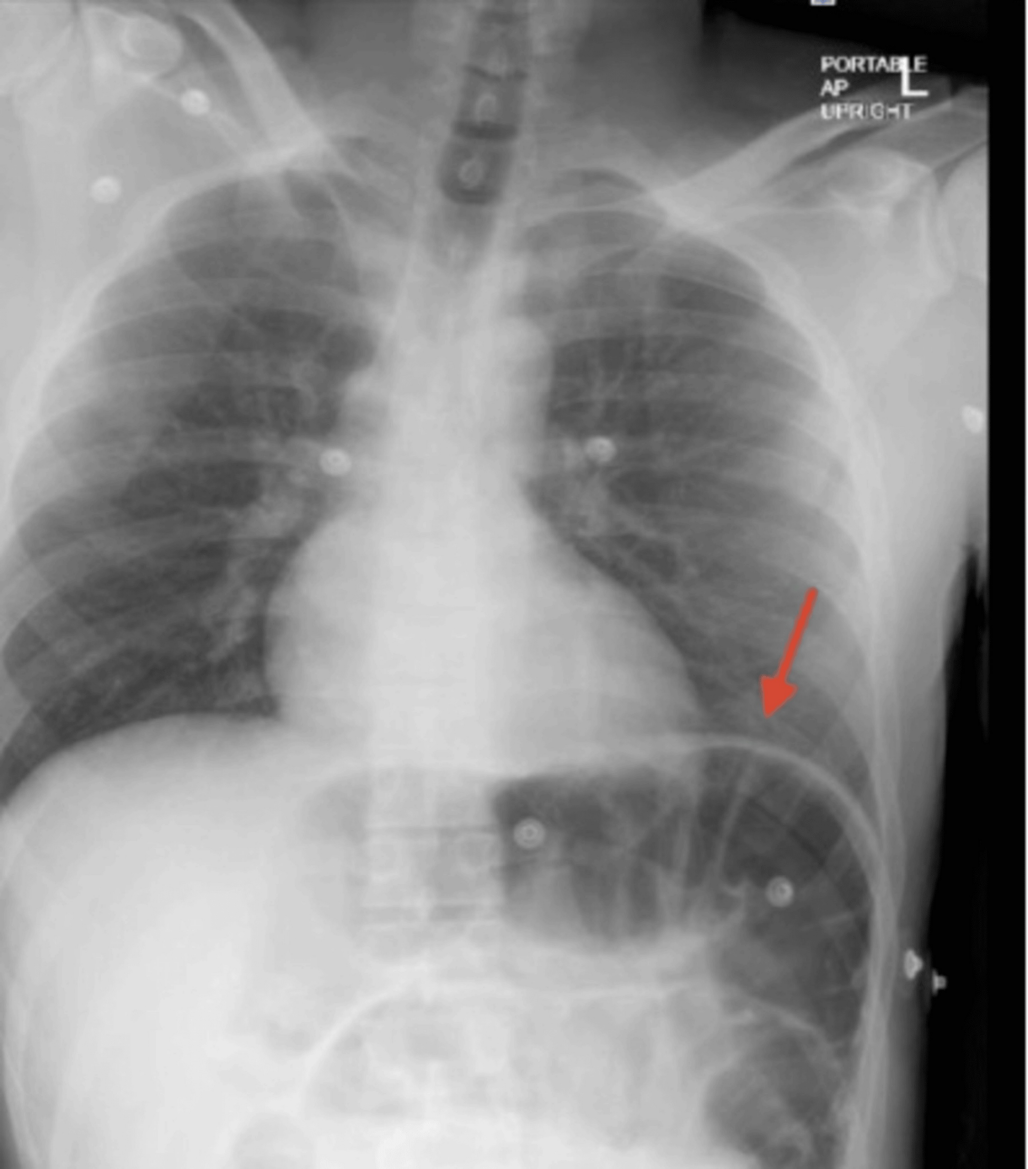
Chest X-ray showing dilation of the bowel The chest X-ray reveals marked gaseous bowel dilation in the left upper quadrant (red arrow), raising concern for an underlying obstruction.

Initial laboratory studies showed an elevated white blood cell count of 16.9/µL (normal range: 4.5-11/µL), but otherwise normal results. Pertinent vital signs included a blood pressure of 126/73 mmHg, a heart rate of 56 beats per minute, and a temperature of 98.6°F. An initial chest X-ray revealed dilated small bowel loops with normal cardiac and pulmonary radiographic findings (Figure 1).

**Figure 2:**
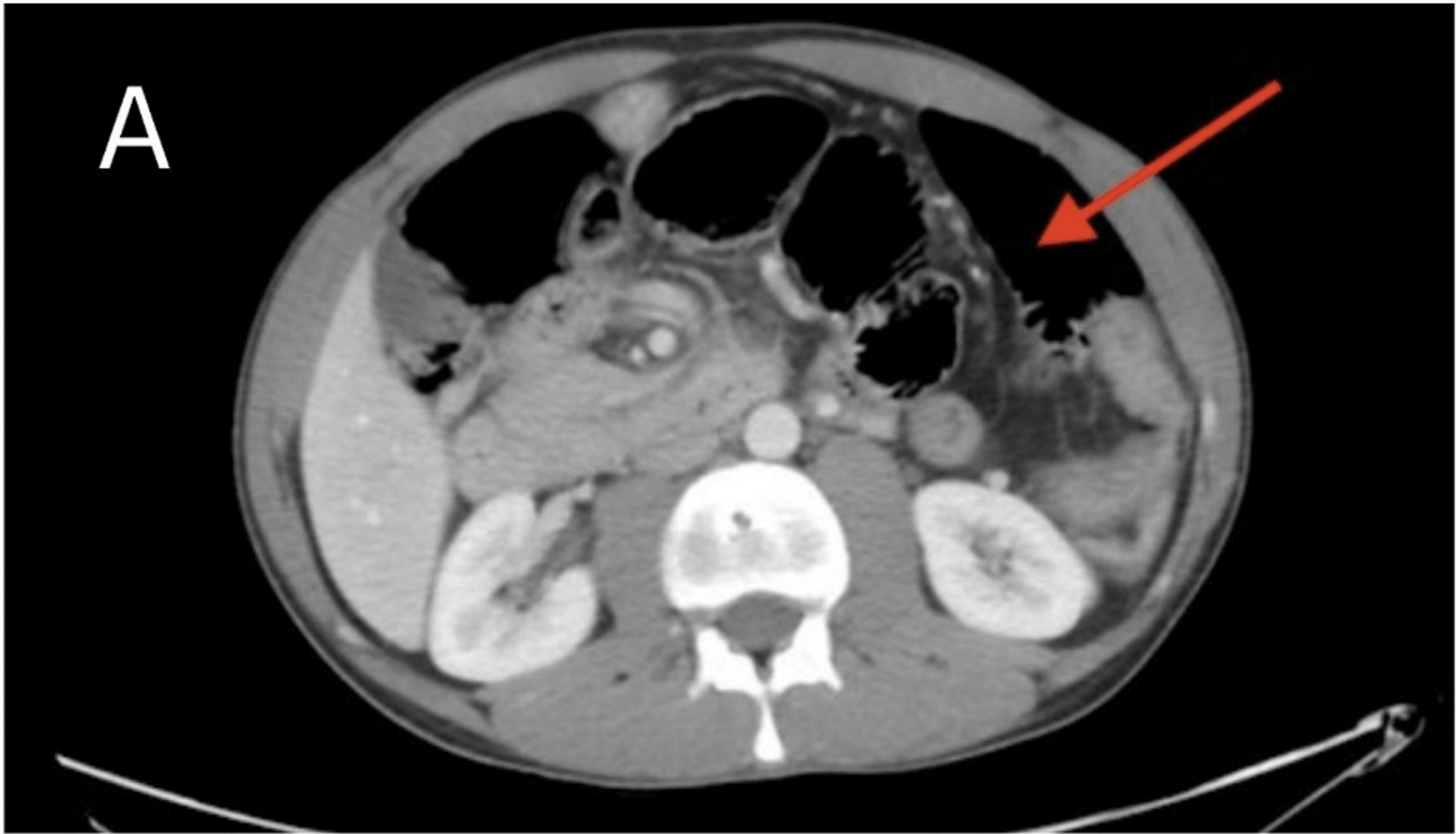
Transverse CT scan of the abdomen The transverse CT image of the abdomen further shows significant dilation of clustered small bowel loops, predominantly in the left upper quadrant, with associated air-fluid levels (red arrow). CT: computed tomography

**Figure 3:**
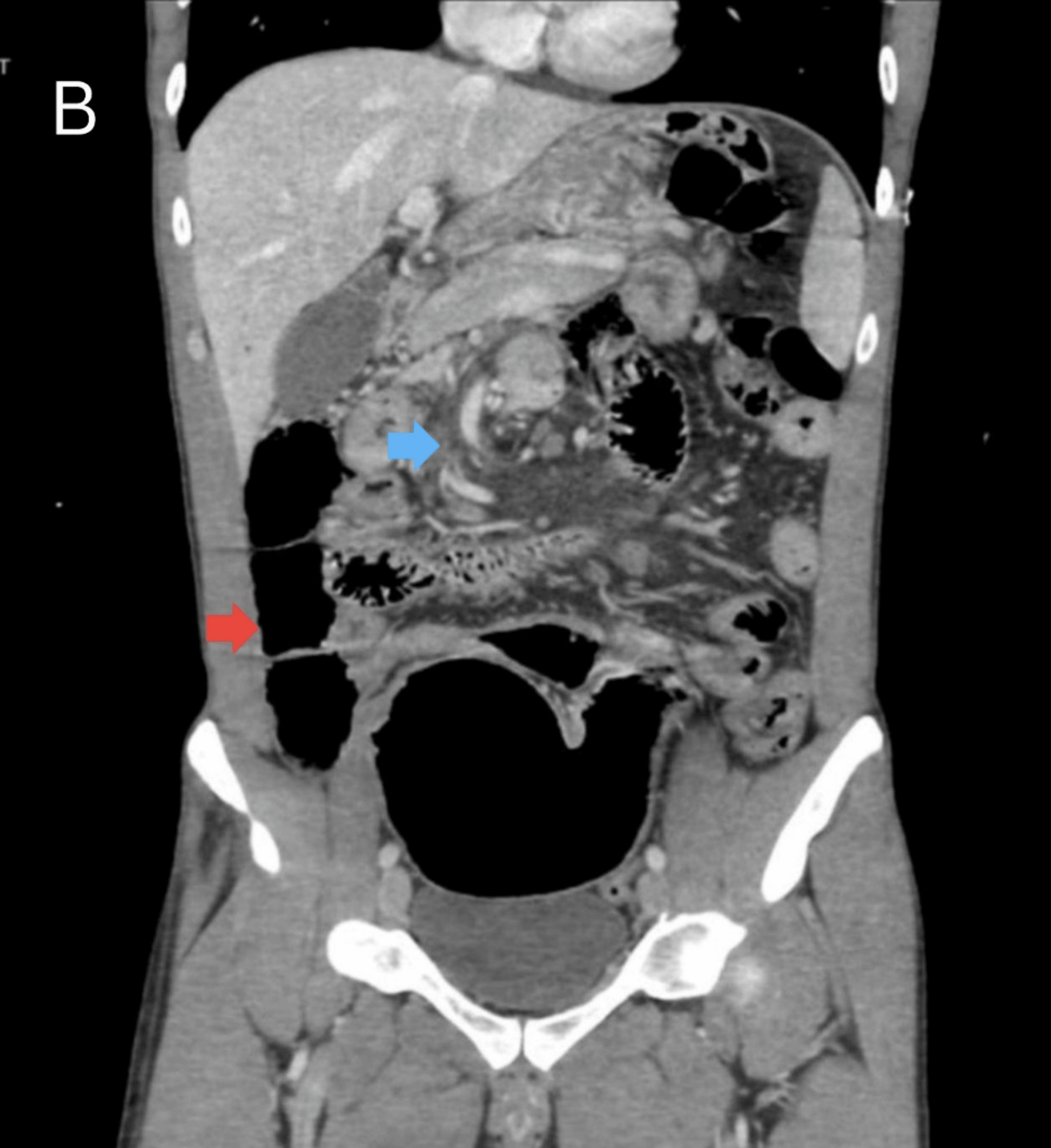
Coronal CT of the abdomen The coronal image of the abdomen further demonstrates diffuse bowel loop distention (red arrow). The configuration of distended bowel loops along with mesenteric crowding and twisting with displacement of vasculature (blue arrow) raises suspicion for a closed-loop obstruction with mesenteric volvulus. CT: computed tomography

A computed tomography (CT) scan of the abdomen and pelvis with intravenous contrast was performed, which demonstrated a cluster of dilated small bowel loops in the left upper quadrant, with the superior mesenteric vessels displaced anteriorly (Figure 2 and Figure 3). While awaiting CT angiography (CTA) for further exploration of possible compromise, the patient's blood pressure dropped to 101/53 mmHg with a concurrent elevation in lactate to 5 mmol/L, prompting fluid resuscitation and emergent surgical evaluation, where the decision was made to take the patient for emergent laparoscopic repair.

The patient was then taken to the operating room, where a laparoscopic exploration was performed. Laparoscopic port placement included Palmer's point at the right subcostal area and two additional ports in the lower right and left quadrants. Upon insufflation and entry into the abdominal cavity, a segment of the small intestine was found herniating through the fossa of Landzert, posterior to the fourth portion of the duodenum. Multiple attempts were made to reduce the hernia laparoscopically, but these proved unsuccessful. As a result, the decision was made intraoperatively to convert to an open procedure, and a midline 10-cm laparotomy incision was created. After entering the peritoneal cavity, the herniated segment of the small bowel was promptly reduced back into its normal position. The superior mesenteric artery (SMA) and the surrounding mesentery were also evaluated for possible vascular compromise, and the SMA was determined to be patent by direct palpation and verified by a Doppler probe. Furthermore, the mesentery surrounding the SMA was directly observed, and no evidence of dusky color or gross ischemic or inflammatory changes was observed.

The entire length of the bowel, from the ligament of Treitz to the cecum, was then carefully inspected, and no signs of ischemia or necrosis were detected. Careful inspection of the fossa of Landzert revealed a defect measuring 5 cm in diameter, which was then closed primarily using running, 2-0 absorbable Stratafix (Ethicon, Raritan, NJ) sutures to ensure closure of the mesenteric defect. The mesentery was carefully inspected for any additional defects, but none were found. The entire length of the small intestine was then re-examined prior to closing the incision. The fascia was closed in a running fashion using an absorbable, monofilament suture material, and the skin was approximated using a subcuticular closure technique, also with an absorbable suture, and the procedure was completed with an estimated blood loss of less than 5 ccs.

The patient had an uncomplicated postoperative course and was discharged on postoperative day 4 without any complications. At a three-month follow-up appointment, the patient reported complete resolution of his abdominal pain and discomfort.

## Discussion

**Figure 4:**
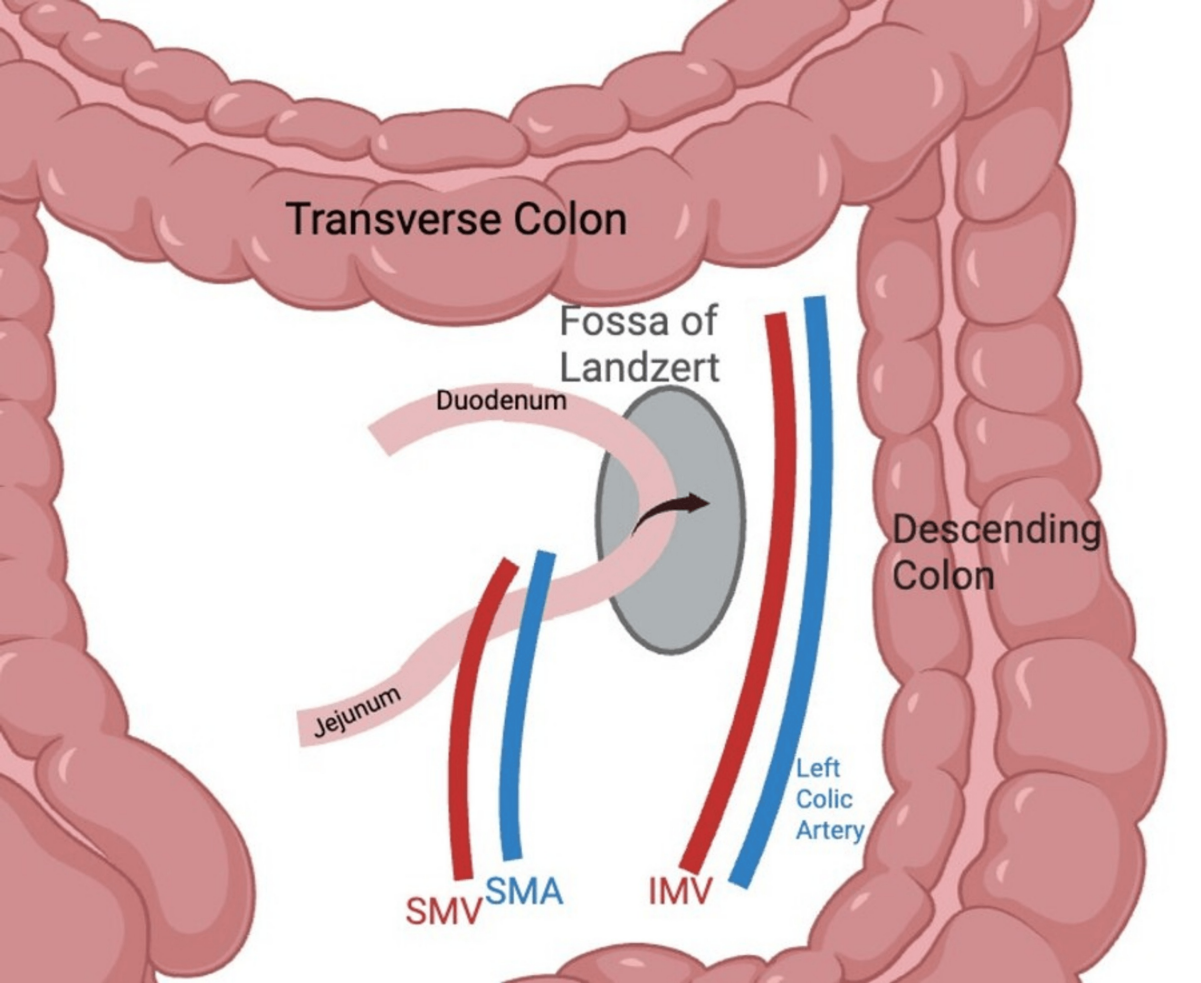
Schematic of Landzert's fossa and bowel herniation A graphic illustration depicting the anatomic defect of Landzert's fossa that predisposes patients to internal paraduodenal herniation. Significant vasculature lies in close proximity to the mesenteric defect, including the left colic artery and IMV laterally and the SMA and SMV medially. Depending on the degree of bowel herniation and potential for volvulus of the hernia sac around its mesenteric root, there is the potential for significant vascular compromise of these vessels. Figure credit: Ishan Deshmukh IMV: inferior mesenteric vein, SMA: superior mesenteric artery, SMV: superior mesenteric vein

Internal hernias, although uncommon, comprising only 0.2%-0.9% of all abdominal wall hernias, can be associated with significant morbidity and mortality if not promptly diagnosed and surgically addressed [1]. Notably, paraduodenal hernias account for roughly half of all internal hernias and may present with a wide range of chronic gastrointestinal symptoms, including intermittent abdominal pain, nausea, vomiting, and post-prandial discomfort [10-12]. These hernias can lead to intermittent intestinal obstruction, further increasing the risk of serious complications if left untreated. In general, the anatomic defect of the fossa of Landzert lies in close proximity to several key vascular structures, particularly the left colic artery and inferior mesenteric vein, as seen in the schematic in Figure 4. Medially to the mesenteric defect lies the superior mesenteric vasculature, which can potentially be obstructed due to the twisting of the hernia sac [2]. The challenging nature of diagnosing paraduodenal hernias is underscored by the rarity of the condition, which can often lead to delayed diagnosis and increased risk of complications.

In this particular case, the patient's initial computed tomography scan of the abdomen revealed significant diffuse edema and displacement of the superior mesenteric artery, which prompted further evaluation with a CTA study. Unfortunately, the patient's condition rapidly deteriorated while waiting for the CTA, necessitating an emergent surgical intervention. During the intraoperative exploration, the patient's bowel and mesenteric tissues were found to have no clear signs of ischemia or necrosis, which was likely due to the spontaneous reduction of the herniated bowel segment prior to the surgery. In this case, the hernia had likely reduced to some degree on its own, leading to the attenuation of the previously observed radiographic findings.

In the present case, the decision was made to proceed with an emergent laparoscopic approach due to the patient's rapidly deteriorating hemodynamic status. While the initial attempts at laparoscopic reduction of the hernia were unsuccessful, the conversion to an open surgical procedure allowed for the prompt reduction of the herniated bowel segment and a careful inspection, which fortunately revealed no evidence of ischemia or necrosis of the bowel. While there are no formal guidelines regarding the use of mesh repair for the surgical management of paraduodenal hernias, in the setting of a paraduodenal hernia with recurrence after primary repair or a large defect or defect that is prone to tension, it might be beneficial to consider utilizing mesh for such cases.

Diagnosing a paraduodenal hernia before surgery offers significant benefits, primarily by facilitating timely and appropriate surgical intervention. Early identification through imaging modalities such as CT scans allows for elective surgical planning, which can prevent complications such as bowel obstruction, ischemia, or necrosis [11]. Moreover, preoperative diagnosis enables surgeons to choose the most suitable surgical approach, potentially favoring minimally invasive techniques that are associated with reduced postoperative pain and quicker recovery times. Recognizing paraduodenal hernia early also aids in avoiding misdiagnosis and ensures that patients receive the correct treatment promptly, with closure of the anatomic defect, thereby improving overall outcomes and preventing future hernia recurrences [12]. When considering whether to begin with a laparoscopic approach or directly starting with an open laparotomy, the size of the hernia should be recognized, as well as the surgical effort and possible complications that go hand in hand with internal hernia repair. For smaller paraduodenal hernias, a laparoscopic approach would be beneficial for hernia reduction and defect closure to improve postoperative outcomes and spare the patient from an open approach; however, if the surgical team anticipates difficulty with a laparoscopic repair, it would be beneficial to utilize an open repair to reduce the amount of time the patient spends on the operating table.

As highlighted in the existing literature, the diagnosis of paraduodenal hernias can be particularly challenging, as these uncommon internal hernias often present with a wide range of nonspecific gastrointestinal symptoms that may initially be attributed to other more common disorders. This can lead to delayed diagnosis and an increased risk of serious complications if the condition is not promptly recognized and surgically addressed.

## Conclusions

Landzert's left paraduodenal hernia is a rare cause of small bowel obstruction that can present with a wide range of nonspecific gastrointestinal symptoms, posing a diagnostic challenge for clinicians. We present this case to expand on the list of potential differential diagnoses for causes of bowel obstruction in patients who are young and without a history of prior abdominal surgery. Prompt recognition and surgical intervention are crucial, as these internal hernias can lead to intestinal volvulus, ischemia, and necrosis if left untreated.
